# Performance of a hybrid capture-based target enrichment next-generation sequencing for the identification of respiratory pathogens and resistance-associated genes in patients with severe pneumonia

**DOI:** 10.1128/spectrum.02130-24

**Published:** 2024-11-19

**Authors:** Wei-Yu Hsu, Ting-Wei Kao, Hsin-Ching Cho, Sheng-Yuan Ruan, Tai-Fen Lee, Yu-Tsung Huang, Jung-Yien Chien, TACTICS (TAiwan CollaboraTive Intensive Care Study) Group

**Affiliations:** 1 Department of Laboratory Medicine, National Taiwan University Hospital, National Taiwan University College of Medicine, Taipei, Taiwan; 2 Department of Internal Medicine, National Taiwan University Hospital, National Taiwan University College of Medicine, Taipei, Taiwan; 3 Department of Laboratory Medicine, National Taiwan University Cancer Center, Taipei, Taiwan; Children's National Hospital, George Washington University, Washington, DC, USA

**Keywords:** FilmArray-PN, pathogen detection, RPIP, severe pneumonia

## Abstract

Severe pneumonia remains the leading infectious cause of death worldwide. The time-consuming nature and suboptimal sensitivity of sputum cultures hamper prompt pathogen detection for tailored treatments. Advanced techniques such as polymerase chain reaction (PCR) and next-generation sequencing (NGS) offer rapid genetic pathogen detection and identification of antimicrobial resistance (AMR) genes. However, the performance of hybrid capture-based target enrichment NGS, e*.*g., Respiratory Pathogen ID/AMR Enrichment Panel (RPIP), for pathogen detection in patients with severe pneumonia remains uncertain. A prospective study involving adults with severe pneumonia was conducted. Respiratory samples from the lower respiratory tract were collected via bronchoalveolar lavage, bronchial washing, or endotracheal tube suction. The performance of RPIP in pathogen and AMR-associated gene detection was compared to that of conventional culture methods and the multiplex PCR-based FilmArray Pneumonia Panel (FilmArray-PN). A total of 83 subjects were enrolled. The most prevalent pathogens detected by RPIP were *Rothia mucilaginosa*, *Stenotrophomonas maltophilia*, *Pseudomonas aeruginosa;* herpes simplex virus-1, cytomegalovirus, and Epstein-Barr virus, and *Pneumocystis jirovecii*. Overall, the positive and negative agreement rates for bacterial detection were 63.6% and 97.5% between RPIP and culture methods, respectively, and 55.8% and 99.4% between FilmArray-PN and culture methods, respectively. Compared to FilmArray-PN, RPIP exhibited significantly better detection rates for bacteria (*P* = 0.029), viruses (*P* < 0.001), and fungi (*P* < 0.001) and identified additional *bla*_OXA_, *bla*_CMY_ as extended-spectrum β-lactamase genes and *bla*_OXA_, *bla*_SHV_ as carbapenemase genes. In conclusion, RPIP can sensitively profile respiratory pathogens and is a promising tool for detecting multiple microorganisms and AMR-associated genes in patients with severe pneumonia.

**IMPORTANCE:**

Sensitive pathogen detection is pivotal for timely treatment by tailoring adequate antimicrobial agents. Unlike conventional phenotypic approach, novel measures using molecular interrogation appear promising. This study aimed to elucidate the efficacy of a hybrid capture-based target enrichment next-generation sequencing technique (Respiratory Pathogen ID/AMR Enrichment Panel, RPIP) as exemplified in a cohort with severe pneumonia. Pathogen landscape in the population was illustrated by these three methodologies. As compared with multiplex polymerase chain reaction-based FilmArray Pneumonia Panel and conventional culture, RPIP demonstrated significantly improved sensitivity in identifying bacteria, viruses, and fungi. The RPIP also exhibited better performance in identifying different pathogens in patients co-infected with multiple microorganisms. Additionally, the genotypes contributing to antimicrobial resistance were determined by RPIP. The study facilitated the implementation of molecular diagnosis by presenting real-world data, whereas future studies are mandated to generalize such an approach toward different clinical settings.

## INTRODUCTION

Severe pneumonia imposes a substantial clinical burden and is the leading infectious cause of death. Epidemiological studies suggested that the annual incidence of severe pneumonia in the intensive care unit is 145 cases per 100,000 adults (1). Respiratory failure, shock, and end-organ failure are common consequences of severe pneumonia. These complications frequently necessitate mechanical ventilation and prolonged intensive care unit stays. Pneumonia developed during ventilator use further compromises the prognosis (2). Additionally, a mortality rate of up to 48% was reported for patients whose condition deteriorated over the course of treatment (3).

Prompt pathogen identification is crucial for guiding antibiotic treatment, particularly in critically ill patients. Appropriate therapy considerably impacts patients’ survival (4). Nevertheless, a previous population-based surveillance of hospitalized individuals with community-acquired pneumonia found that only 38% of the patients exhibited positive pathogen detection results in blood, sputum, or urine samples, with the most identified pathogens being viruses (5). In addition to its suboptimal sensitivity, sputum culture is time-consuming, which can result in delays in the administration of appropriate anti-microbiological agents. The morphological diagnostic modalities relied on adequate clonal expansion of the pathogens. Either unviable species due to prior anti-microbiological agent use or suboptimal proliferation on the cultural niche resulted in a delayed and insensitive diagnosis of respiratory pathogens. Consequently, alternative methodologies that transition from morphological to molecular diagnosis have emerged. Reverse transcription polymerase chain reaction (PCR)-based approaches have shown promise for rapid pathogen detection (6). Unlike culture-based methods, PCR does not require viable pathogens for detection and is less affected by prior antimicrobial treatments (7). The positive results can be obtained purely based on the presence of genomic segments from respiratory pathogens. The BioFire FilmArray Pneumonia Panel (FilmArray-PN) (8) exemplifies the application of multiplex PCR for genotypic detection and has been validated in a cohort with lower respiratory tract infections (9). However, this method could identify only pathogens with a predetermined spectrum of gene segments. Even though mitochondrial small subunit rRNA gene-targeted MultiCode PCR assay has been established to detect *Pneumocystis jirovecii* (10), several species, including *Legionella pneumophila* and *Mycoplasma pneumonia*, could not be identified, highlighting a clinically unmet need.

Metagenomic next-generation sequencing (NGS) approach appears promising for complementing this detection gap by analyzing either a targeted amplicon assay or untargeted shotgun sequencing. Accumulating literature addressed the role of NGS especially with re-emerging neutropic viruses and disease outbreaks (11, 12). More than 280 respiratory pathogens and >1,200 antimicrobial resistance (AMR) genotypes can be identified by metagenomic NGS using the Respiratory Pathogen ID/AMR Enrichment Panel (RPIP) Kit (13). A previous study also reported a markedly higher positive rate of bacterial recognition (14). However, there is a paucity of real-world data available to demonstrate metagenomic NGS performance. Additionally, no prior study has directly compared the diagnostic performance of RPIP, FilmArray-PN, and culture-based techniques. This study aimed to elucidate the efficacy of these three measures in a cohort of patients with severe pneumonia.

## RESULTS

### Cohort characteristics

A total of 83 patients with severe pneumonia were included in this study. The mean age of the cohort was 67.6 years old, and 60.2% of the patients were male. Most patients were critically ill, with an intensive care unit admission rate of 85.5% and a high in-hospital mortality rate of 48.2%. The average CURB-65 score was 2.2. Notably, 71.1% of the cohort were intubated, and 6.02% required venovenous extracorporeal membrane oxygenation. Although the presence of neurological, cardiac, metabolic, hepatic, nephrotic, and autoimmune comorbidities as well as underlying pulmonary diseases was low, there was a high prevalence of both hematological malignancies (36.1%) and solid tumors (36.1%). The samples of lower respiratory tract were harvested mostly from suction through an endotracheal tube or tracheostomy, with the remaining from bronchoalveolar lavage or bronchial washing (Table S1). Detailed demographic and clinical parameters are summarized in Table 1.

**TABLE 1:** Demographic and clinical parameters of included cohort^*a*^

Characteristics (*N* = 83)	Data
Age	67.6 ± 15.9
Male	50 (60.2%)
Disease severity	
ICU admission	71 (85.5%)
In-hospital mortality	40 (48.2%)
CURB-65	2.2 ± 1.3
O_2_ demand	
FiO_2_	45.3% ± 20.0%
MV + VV-ECMO	5 (6.02%)
MV	54 (65.1%)
NIPPV	1 (1.20%)
High-flow nasal cannula	5 (6.02%)
Mask	1 (1.20%)
Nasal cannula	10 (12.0%)
Ambient air	8 (9.64%)
Comorbidity	
Stroke	15 (18.1%)
Dementia	8 (9.64%)
Heart failure	11 (13.3%)
Coronary artery disease	15 (18.1%)
Valvular heart disease	5 (6.02%)
Diabetes mellitus	23 (27.7%)
Hypertension	33 (39.8%)
Chronic kidney disease	13 (15.7%)
Liver cirrhosis	3 (3.61%)
Chronic obstructive pulmonary disease	5 (6.02%)
Asthma	3 (3.61%)
Intestinal lung disease	4 (4.82%)
Autoimmune disease	9 (10.84%)
Hematological malignancy	30 (36.1%)
Solid tumor	30 (36.1%)

^*a*^ ICU, intensive care unit; MV, mechanical ventilation; VV-ECMO, venovenous extracorporeal membrane oxygenation; NIPPV, noninvasive positive pressure ventilation.

### Pathogen detection rate

**Fig 1:**
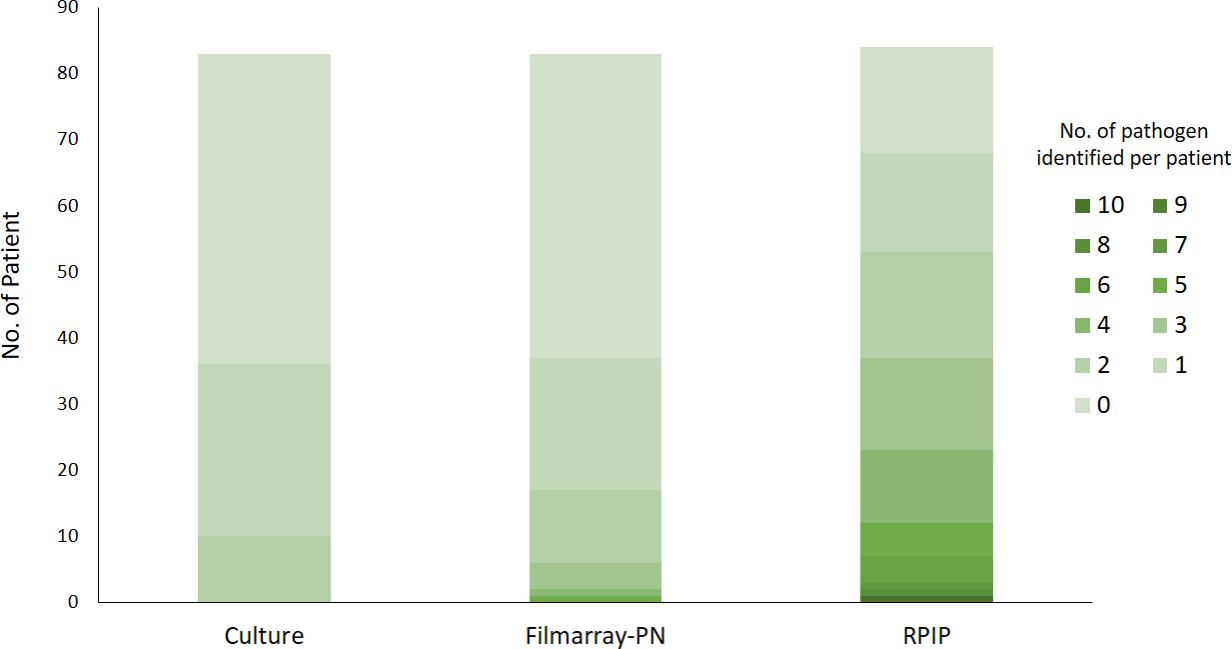
Number of pathogens detected by culture, FilmArray-PN, and RPIP.

**Fig 2:**
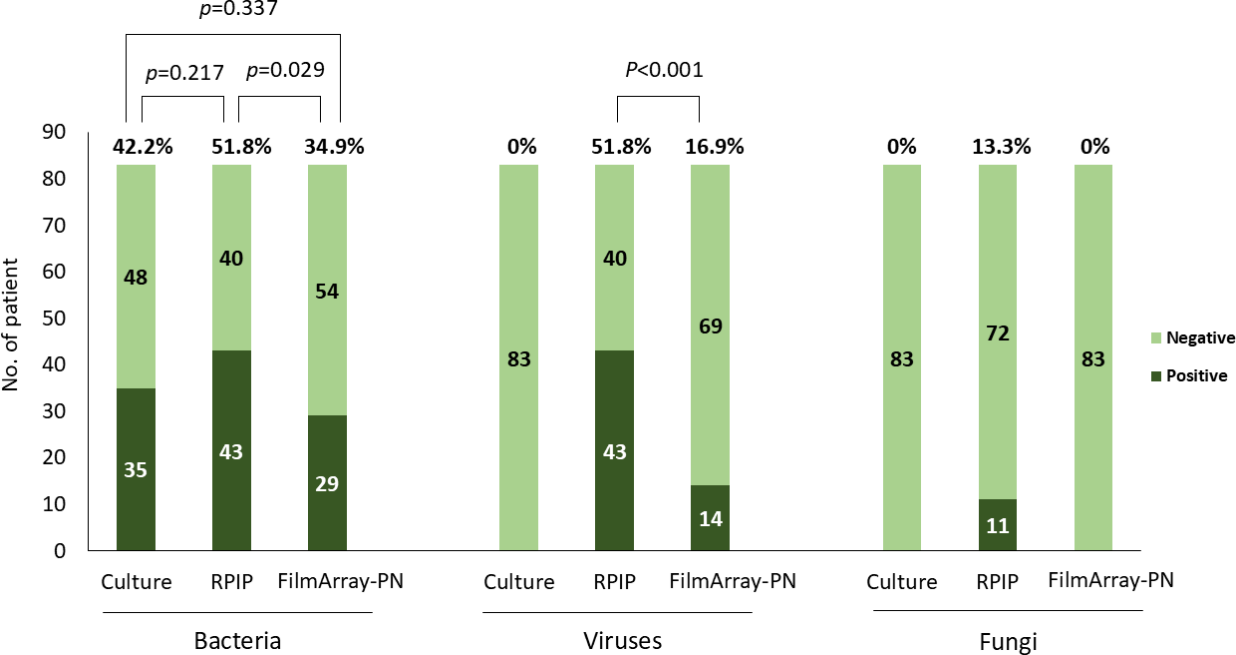
Positivity rate comparison between culture, FilmArray-PN, and RPIP.

Initially, the diagnostic performance of three methodologies was compared. For quantitative analysis, a total of 67, 37, and 36 pathogens were identified from the cohort using RPIP, FilmArray-PN, and culture methods, respectively. RPIP was particularly sensitive in detecting multiple pathogens in a single individual (Fig. 1). Significantly higher rates of bacteria were detected by RPIP (66.3%) than by FilmArray-PN (49.4%) (*P* = 0.029). Nevertheless, there were no statistically significant differences between RPIP/culture methods (*P* = 0.217) and FilmArray-PN/culture methods (*P* = 0.337). The detection rates for viruses and fungi by RPIP were remarkably higher than those by either culture methods or FilmArray-PN (Fig. 2).

**Fig 3:**
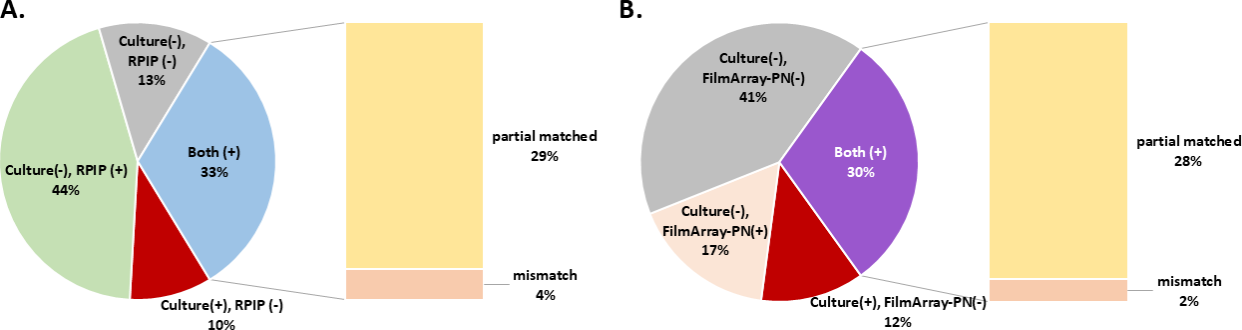
Positive rate agreement between culture and (**A**) RPIP and (**B**) FilmArray-PN.

An agreement analysis for these techniques was also performed (Table 2). The positive percentage agreement for culture/RPIP reached 63.6% for bacteria, with a higher rate observed for Gram-positive species (80.0%) than for Gram-negative species (61.5%). The negative percentage agreement remained high across bacteria, viruses, and fungi. A similar pattern was observed for the culture/FilmArray as well. The positive and negative percentage agreements for each pathogen are shown (Table S2). For RIPP and culture methods, 27 patients (33%) were identified as being positive for pathogens detected by both techniques; the detected pathogens were partially matched in 24 participants and completely mismatched in 3. A positive RPIP yield but negative culture result was observed in 37 patients (44%), whereas 8 patients (10%) were rendered positive by culture but negative by RPIP. Eleven patients (13%) tested negative using both the techniques. In terms of FilmArray-PN and culture methods, positive pathogens were detected by both the techniques in 25 patients (30%), including 23 individuals (28%) exhibiting partially matched pathogens and 2 individuals (2%) with mismatched pathogens. Fourteen patients (17%) were rendered positive by FilmArray-PN but not by culture methods, whereas positive culture but negative FilmArray-PN results were observed in 10 patients (12%). Thirty-four participants (41%) were found to be negative for pathogens using both techniques (Fig. 3).

**TABLE 2:** Positivity rate comparison and agreement analysis^*a*^

	Culture (+) RPIP (+)	Culture (+) RPIP (−)	Culture (−) RPIP (+)	Culture (−) RPIP (−)	PPA % (CI)	NPA % (CI)
No. of bacterium	28	16	96	3,678	63.6 (47.8–77.6)	97.5 (96.9–97.9)
Gram-positive	4	1	32	876	80.0 (28.4–99.5)	96.5 (95.1–97.6)
Gram-negative	24	15	64	2,802	61.5 (44.6–76.6)	97.8 (97.2–98.3)
No. of virus	0	0	65	765	NA	92.2 (90.1–93.9)
dsDNA virus	0	0	50	365	NA	88.0 (84.4–90.9)
RNA virus	0	0	15	400	NA	96.4 (94.1–98.0)
No. of fungus	0	0	11	321	NA	96.7 (94.1–98.3)

	Culture (+) FilmArray-PN (+)	Culture (+) FilmArray-PN (−)	Culture (−) FilmArray-PN (+)	Culture (−) FilmArray-PN (−)	PPA % (CI)	NPA % (CI)
No. of bacterium	24	19	24	3,751	55.8 (39.9–70.9)	99.4 (99.1–99.6)
Gram-positive	5	1	6	901	83.3 (35.9–99.6)	99.3 (98.6–99.8)
Gram-negative	19	18	18	2,850	51.4 (34.4–68.1)	99.4 (99.0–99.6)
No. of virus	0	0	14	816	NA	98.3 (97.2–99.1)
dsDNA virus	0	0	3	412	NA	99.3 (97.9–99.9)
RNA virus	0	0	11	404	NA	97.3 (95.3–98.7)
No. of fungus	0	0	0	332	NA	100.0 (98.9–100.0)

^*a*^ RPIP, Respiratory Pathogen ID/AMR Enrichment Panel Kit; FilmArray-PN, BioFire FilmArray Pneumonia Panel; PPA, positive percentage agreement; NPA, negative percentage agreement; CI, confidence interval; NA, not applicable.

### Pathogen landscape

**Fig 4:**
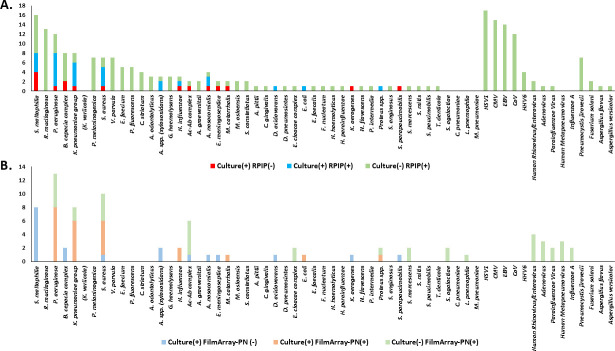
Pathogen landscape detected by (**A**) RPIP and (**B**) FilmArray-PN, stratified by culture results. HSV-1, herpes simplex virus-1; CMV, cytomegalovirus; EBV, Epstein-Barr virus; CoV, coronavirus; HHV-6, human herpesvirus 6.

The pathogen landscape was also investigated (Fig. 4). The detection rate of any bacterium was 66.3% by RPIP and 49.4% by FilmArray-PN. The most commonly identified bacteria by RPIP were *Rothia mucilaginosa* (15.7%), *Stenotrophomonas maltophilia* (14.5%), and *Pseudomonas aeruginosa* (13.3%), whereas the FilmArray-PN commonly identified *P. aeruginosa* (15.7%), *Klebsiella pneumoniae* (9.6%), and *Staphylococcus aureus* (10.8%). Only one patient was positive for atypical bacteria, *L. pneumophila* detected by FilmArray-PN, but not by RPIP or culture methods. *Mycoplasma pneumoniae* and *C. pneumoniae* were not identified by either technique. By RPIP, the detection rate of any virus was 55.4%, while the bacterial and viral coinfection rate was 51.8% of total population. The most prevalent viral pathogens detected by RPIP were herpes simplex virus-1 (19.3%), cytomegalovirus (18.1%), Epstein-Barr virus (16.9%), severe acute respiratory syndrome coronavirus 2 (SARS-CoV-2) (14.5%), and human herpesvirus 6 (4.8%). However, these pathogens were not detected by FilmArray-PN. The fungi *P. jirovecii* (8.4%) and *Aspergillus* (2.4%) were the dominant pathogens identified using RPIP. Seven patients were also rendered *P. jirovecii*-positive by RPIP, of which five (71.4%) showed concordant outcomes by PCR. The detailed numbers of detected pathogens are summarized in Table 3.

**TABLE 3:** Total numbers of pathogen detected (*N* = 83)^*a*^

	RPIP	FilmArray-PN
Any bacterium	55 (66.3%)	41 (49.4%)
*Rothia mucilaginosa*	13 (15.7%)	NA
*Stenotrophomonas maltophilia*	12 (14.5%)	NA
*Pseudomonas aeruginosa*	11 (13.3%)	13 (15.7%)
*Burkholderia cepacia complex*	6 (7.2%)	NA
*Klebsiella pneumoniae group (variicola*)	7 (8.4%)	8 (9.6%)
*Prevotella melaninogenica*	7 (8.4%)	NA
*Staphylococcus aureus*	6 (7.2%)	9 (10.8%)
*Veillonella parvula*	7 (8.4%)	NA
*Enterococcus faecium*	5 (6.0%)	NA
*Pseudomonas fluorescens*	5 (6.0%)	NA
Any virus	46 (55.4%)	NA
Herpes simplex virus-1	16 (19.3%)	
Cytomegalovirus	15 (18.1%)	
Epstein-Barr virus	14 (16.9%)	
SARS-CoV-2	12 (14.5%)	
Human herpesvirus 6	4 (4.8%)	
Any fungi	10 (12.0%)	NA
*Pneumocystis jirovecii*	7 (8.4%)	
*Aspergillus*	2 (2.4%)	
Drug resistance		
Any resistance genes	8 (9.6%)	9 (10.8%)
ESBL	6 (7.2%)	6 (7.2%)
*bla*_CTX-M_	4 (4.8%)	6 (7.2%)
*bla*_OXA_	1 (1.2%)	NA
*bla*_CMY_	1 (1.2%)	NA
Carbapenemases	5 (6.0%)	6 (7.2%)
*bla*_OXA_	3 (3.6%)	NA
*bla*_SHV_	2 (2.4%)	NA
*bla*_IMP_	1 (1.2%)	5 (6.0%)
*bla*_NDM_	1 (1.2%)	1 (1.2%)
Methicillin resistance (*mec A/C* & *MREJ*)	0	1 (1.2%)

^*a*^ ESBL, extended-spectrum β-lactamases; RPIP, Respiratory Pathogen ID/AMR Enrichment Panel Kit; FilmArray-PN, BioFire FilmArray Pneumonia Panel; NA, not applicable.

### AMR genotyping

AMR were identified upon several species of bacteria, including *K. pneumoniae* (30.8%), *P. aeruginosa* (30.8%), *Acinetobacter nosocomialis* (15.4%), *Escherichia coli* (6.3%), *Proteus mirabilis* (6.3%), and *S. aureus* (6.3%). Notably, a total of 11 patients with considerable discrepancies in AMR were observed between the cultures, RPIP, and FilmArray-PN techniques (Table 4). Three out of four positive bacterial cultures with notable drug resistance were detected for AMR by RPIP or FilmArray-PN; however, the consistency rate of AMR categories between RPIP and FilmArray-PN was only 30.8%. The AMR genotypes depicted by RPIP or FilmArray-PN were *bla*_CTX-M_, *bla*_OXA_, and *bla*_CMY_ for extended-spectrum β-lactamases, *bla*_OXA_, *bla*_SHV_, *bla*_IMP_, and *bla*_NDM_ for carbapenemases, and *mec A/C & MREJ* for methicillin-resistance.

**TABLE 4:** Genetics of drug resistance^*a*^^,^^*b*^

	Bacterial culture	Resistance gene by FilmArray-PN	Resistance gene by RPIP	Drug sensitivity test results (minimum inhibitory concentration)																
				AN	CAZ	CIP	CL	FEP	GM	ETP	IPM	LVX	MEM	SAM	TGC	TZP	CMZ	CTX	SXT	CZ
86/M	*Acinetobacter nosocomialis*	Negative	***blaO*_XA-444_ (carbapenemase**)		S (8)	S (≤0.25)	I (≤0.5)	S (2)	S (≤1)		S (≤0.25)	S (≤0.12)	S (≤0.25)	S (≤2)	(≤0.5)	S (≤4)				
80/F	*Pseudomonas aeruginosa*	*bla*_IMP_ (carbapenemases)	*bla*_IMP-8_ (carbapenemases)	S (≤2)	S (4)	I (1)	I (2)	S (2)	S (≤1)		S (2)	R (4)	S (1)		(≥8)	S (8)				
74/M	*Pseudomonas aeruginosa*	Negative	***bla*_OXA-50_ (ESBL**)	S (≤2)	S (8)	S (≤0.25)	I (2)	S (4)	S (≤1)		R (≥16)	S (1)	R (≥16)		(≥8)	I (32)				
68/F	*Pseudomonas aeruginosa*	***bla*_IMP_ (carbapenemases**)	Negative	S (≤2)	S (2)	S (≤0.25)	I (2)	S (2)	S (≤1)		S (1)	S (1)	S (≤0.25)		(≥8)	S (8)				
	*Acinetobacter nosocomialis*	***bla*_IMP_ (carbapenemases**)	Negative		S (4)	S (≤0.25)	I (2)	S (2)	S (≤1)		S (≤0.25)	S (≤0.12)	S (≤0.25)	S (≤2)	(≤0.5)	S (≤4)				
69/M	*Pseudomonas aeruginosa*	***bla*_IMP_** (**carbapenemases**)	Negative	S (4)	R (≥64)	S (≤0.25)	I (2)	R (≥64)	S (2)		R (≥16)	S (1)	R (8)		(≥8)	R (≥128)				
62/F	*Klebsiella pneumoniae*	*bla*_CTX-M_ (ESBL) + *bla*_OXA-48-like_ (carbapenemases)	*bla*_CTX-M-14_ (ESBL) + *bla*_OXA-181_ + *bla*_SHV-12_ (carbapenemase)	S (≤2)	R (≥64)	R (≥4)	I (2)	SDD (8)	R (≥16)	R (≥8)	S (1)	R (≥8)	S (1)	R (≥32)	R (≥8)	R (≥128)	S (16)	R (≥64)	R (≥320)	R (≥64)
62/M	*Escherichia coli*	*bla*_CTX-M_ (ESBL)	*bla*_CTX-M-55_ (ESBL)	S (≤2)	R (≥64)	S (≤0.25)	R (≥16)	R (≥64)	S (≤1)	S (≤0.5)	S (≤0.25)	S (≤0.12)	S (≤0.25)	R (≥32)	S (2)	R (64)	S (4)	R (≥64)	R (≥320)	R (≥64)
60/M	*Klebsiella pneumoniae*	*bla*_CTX-M_ (ESBL)	*bla*_CTX-M-15_ (ESBL) + **blaOXA_OXA-1_ + blaSHV_SHV-1_ (carbapenemases**)	S (≤2)	R (≥64)	R (≥4)	I (≤0.5)	R (≥64)	R (≥16)	R (≥8)	S (≤0.25)	R (≥8)	S (1)	R (≥32)	S (2)	R (≥128)	R (≥64)	R (≥64)	R (≥320)	R (≥64)
64/M	*Klebsiella pneumoniae*	***bla*_CTX-M_ (ESBL**)	Negative	S (4)	R (≥64)	R (≥4)	I (≤0.5)	R (≥64)	S (≤1)	S (≤0.5)	S (0.5)	R (≥8)	S (≤0.25)	R (≥32)	S (2)	R (≥128)	S (≤1)	R (≥64)	S (≤20)	R (≥64)
84/M	*Klebsiella pneumoniae*	*bla*_CTX-M_ (ESBL) + *bla*_NDM_ + *bla*_VIM_ (carbapenemases)	*bla*_CTX-M80_ (ESBL) + *bla*_NDM-24_ (carbapenemases)	S (≤2)	S (≤1)	S (≤0.25)	I (≤0.5)	S (≤1)	S (≤1)	S (≤0.5)	S (≤0.25)	I (1)	S (≤0.25)	I (16)	I (4)	SDD (16)	S (2)	S (≤1)	S (≤20)	S (≤4)
85/F	*Proteus mirabilis*	***bla*_CTX-M_** (ESBL)	***bla*_CMY-2_** (ESBL)	S (≤2)	S (4)	R (2)		S (≤1)	I (≤8)	S		R (4)	S (0.5)	R (≥32)		S (≤4)				
	*Staphylococcus aureus*	***mec A/C*** *& **MREJ*** (**MRSA**)	Negative	OX	VA	CIP	E	CC	GM	TE	FA	LVX	SXT	LZD	DAP					
				R (≥4)	S (≤0.5)	S (≤0.5)	R (4)	R (0.5)	I (8)	R (≥16)	S (≤0.5)	S (≤0.12)	S (≤10)	S (2)	S (0.25)					

^*a*^ RPIP, Respiratory Pathogen ID/AMR Enrichment Panel Kit; FilmArray-PN, BioFire FilmArray Pneumonia Panel; AN, amikacin; CAZ, ceftazidime; CIP, ciprofloxacin; CL, colistin; FEP, cefepime; GM, gentamicin; IPM, imipenem; LVX, levofloxacin; MEM, meropenem; SAM, ampicillin/sulbactam; TGC, tigecycline; TZP, pipera/tazobactam; CMZ, ceftriaxone; CTX, cefotaxime; SXT, sulfamethoxazole-trimethoprim; CZ, cefazolin; OX, oxacillin; VA, vancomycin; E, erythromycin; CC, clindamycin; TE, tetracycline; FA, fusidic acid; LZD, linezolid; DAP, daptomycin; S, susceptible; I, intermediate; R, resistant; SDD, susceptible-dose dependent.

^*b*^ Words with boldface indicate the different genotypes identified by FilmArray-PN or RPIP.

## DISCUSSION

In this prospective cohort study, we characterized the diagnostic performance of RPIP, FilmArray-PN, and culture-based techniques in patients with severe pneumonia. To our knowledge, this is the first study to directly compare the yield rate of pathogen detection among these three techniques. The major findings are as follows: (i) RPIP exhibited better efficiency and sensitivity in identifying respiratory pathogens, demonstrating a better yield rate than both FilmArray-PN and culture methods. (ii) Viruses and fungi were most likely to be detected by RPIP but not by FilmArray-PN or culture methods. (iii) Positive agreement rates remained moderate, whereas negative agreement rates were high between RPIP/culture and FilmArray-PN/culture methods. (iv) RPIP identified pathogens with drug resistance and determined specific AMR genotypes as well.

Severe pneumonia necessitates prompt administration of antimicrobial agents, but drug choice remains largely empirical and dependent on risk factors (15). An increasing trend in genotypic approaches has contributed to earlier and more sensitive detection of pathogens. Previous studies have validated the utility of NGS-based measures in cohorts with various clinical contexts, including pneumonia (16) and sepsis, and have analyzed samples collected from different sites. Additionally, NGS-based techniques have contributed to the identification of severe acute respiratory syndrome coronavirus 2 during early outbreaks (17). Moreover, integration of NGS-based metagenomics facilitates infection treatment, resulting in better outcomes (18). However, the detection rate of fungi remains low using NGS, which has been identified as a major limitation (19). Further refinements of sample extraction, library establishment, and sequencing techniques are required to improve sensitivity.

Coinfection with bacteria, viruses, and fungi in patients with severe pneumonia is common and is correlated with a worse prognosis (20). An advantage of the NGS-based technique is that it is effective in characterizing concurrent infections by different pathogens. In our cohort with positive culture outcomes, more than half of the individuals were identified as having multiple pathogens using RPIP. The lung is not a sterile niche, and studies have demonstrated that the presence of certain pathological species often represents colonization without causing harm rather than active infection (21). Nevertheless, the clinical significance of these additionally identified pathogens remains undetermined; therefore, the interpretation of NGS-based outcomes should be prudent. Whether these additionally recognized microorganisms mandate corresponding treatments or should be regarded as mere bystander colonization remains a matter of clinical judgment. Accurate phenotyping of microorganisms identified by RPIP into categories such as normal flora, those associated with respiratory diseases, and pathogenic species may promote treatment guidance (22). The development of a deep-learning algorithm, assisted by artificial intelligence, to accurately identify true pathogens is a promising future perspective (23).

In this study, the pathogenic landscape of a cohort of patients with severe pneumonia was delineated. Although *Streptococcus pneumoniae* has long been recognized as the leading bacterial culprit (24), its prevalence was low, as identified by both genotypic methods in our study. For virus diagnostics, the result of RPIP was similar to a previous metagenomic study that identified herpes as the most prevalent (25). However, FilmArray-PN detected more rhinovirus/enterovirus infections. These discrepancies may be attributed to the predefined probes used in PCR, instead of the hypothesis-free approach used in NGS. Notably, our cohort included a substantial proportion of patients with hematological malignancies, and their potentially immunocompromised status could have altered the prevalent pathogen identified. Moreover, cytomegalovirus is known to predominate in such populations (26) and RPIP coherently identified multiple pathogens associated with opportunistic infections in our cohort. However, determining the optimal timing for genotypic pathogen detection remains a clinical challenge. Expert opinions suggest that metagenomic NGS should be considered for immunocompromised patients with positive conventional tests but experiencing treatment failure, or for those with negative conventional tests who continue to exhibit signs of infection (27). For immunocompetent patients, a second set of conventional examinations is recommended before the initiation of metagenomic NGS.

Unlike phenotypic antibiotic sensitivity testing, the genotypic approach addresses selected resistance mechanisms (28). RPIP is particularly sensitive to identifying specific AMR genotypes. The prevalence of drug-resistant pathogens was unexpectedly low in our cohort, and the distribution of different AMR genes also varied across species. NGS-based approaches have revolutionized the detection of AMR, and studies have documented how NGS facilitates global AMR surveillance (29) to help in tracking the outbreak of AMR pathogens (30) from a public health perspective. Similar to the abundance of microorganisms identified, the abundance of readouts generated from AMR genetics mandates the use of integrated bioinformatics tools for rapid real-time analysis (31).

Our study has several limitations. First, it was descriptive in nature and did not correlate the pathogen landscape or AMR genetics with clinical outcomes. Potential confounding factors included most elderly patients, high prevalence of hematological malignancies and solid cancers, as well as single ethnicity background. Therefore, the interpretation of the results must be prudent, and generalization to other populations mandates more investigations. Future studies are also warranted to identify prognostic factors in individuals with severe pneumonia and certain microbiological-resistant or drug-resistant phenotypes as well. Secondly, the specimen subjected to RPIP and FilmArray-PN was not identical to those used for culture. Although the samples were collected from the lower respiratory tract of the same patients at similar time points, the use of different specimens may introduce discrepancies in results. Third, these patients underwent respiratory pathogen identification only once during their admission. Temporal changes, along with anti-microbiological agent treatment and the serial predominant pathogens during the clinical trajectory, remain enigmatic. Fourth, only patients with severe pneumonia were included in the study. Whether the yield rates of RPIP will be better maintained in individuals with less disease burden than in those with critical status requires future cohort studies. Fifth, the sensitivity of several respiratory pathogens which are mostly detected in pediatrics, including enterovirus and human parechoviruses, remained unknown due to limited sample size and predominantly elderly patients included in this study. Previous study using EliTech HPeV RT-PCR with universal primers appeared promising and can potentially be applied in different population for pathogen detection (32, 33).

In conclusion, RPIP is sensitive for pathogen detection, especially for viruses, fungi, and patients co-infected with multiple microorganisms. RPIP further facilitates the delineation of AMR genotypes, as exemplified in individuals with severe pneumonia. The pathogen landscape investigated by RPIP in our cohort may be used as a reference to tailor future empirical antimicrobial agent choices.

## MATERIALS AND METHODS

### Study cohort

The cohort with severe pneumonia was prospectively enrolled from June to December 2023 at two medical institutions (National Taiwan University Hospital, Taipei, Taiwan and National Taiwan University Cancer Center, Taipei, Taiwan). Adults (>20 years of age) with clinically diagnosed severe pneumonia were included if they met one of the following criteria: (i) oxygen saturation <90% under ambient air, (ii) development of acute respiratory distress syndrome, or (iii) pneumonia necessitating intubation, bronchoalveolar lavage, or bronchial washing. Only participants who underwent all three tests (RPIP, FilmArray-PN, and culture-based studies) were included. Disease severity was assessed using the intensive care unit admission rate, in-hospital mortality rate, and CURB-65 score. The oxygen demand and oxygen delivery devices were documented at the time when respiratory tract samples were collected. Other comorbidities were clinically evaluated.

### Sample harvest

Lower respiratory tract samples were obtained within 24 h of diagnosing severe pneumonia. Specimens were exclusively collected via bronchoscopy or sputum suction through an endotracheal tube/tracheostomy. Each sample was processed using both FilmArray-PN and RPIP to investigate the presence of microorganisms and AMR genotypes. Conventional cultures and antibiotic sensitivity tests were performed on different respiratory samples collected from the same individual.

### Sample processing

For identification of bacterial cultures, respiratory tract specimens were inoculated on a blood agar/eosin methylene blue agar biplate (Becton, Dickinson Microbiology Systems) and chocolate agar. The samples were subsequently incubated in an enriched environment with 5% CO_2_ at 35°C, and observed after 18–24 h. Cultures were monitored for an additional 4 days before reporting negative results. For identification of viruses, the samples were cultured with HeLa, human epithelial cell-2, rhabdomyosarcoma, LLC-rhesus monkey kidney cell-2, and Mardin-Darby canine kidney cells for a maximum of 28 days. The fungi were cultured on Sabouraud dextrose agar, Mycocel slants, and inhibitory mold agar slants (Becton, Dickinson Microbiology Systems) for 28 days. A VITEK 2 system (bioMérieux, Marcy l'Etoile, France) was used for antibiotic sensitivity testing (34).

The PCR-based pathogen detection was performed according to the manufacturer’s instructions for the “BioFire FilmArray Pneumonia Panel.” Respiratory tract specimens were inoculated using a flocked swab into the FilmArray-PN injection vial containing sample buffer. The vial was then capped and inverted to facilitate microorganism release before being transferred into a test pouch for analysis, which lasted for approximately 75 min. The pouch contained all the reagents required for sample lysis, nucleic acid extraction, reverse transcription, amplification, and detection. A total of 33 genomic sequences of panel targets are available through the FilmArray-PN. For bacterial targets, the FilmArray-PN yielded a log10 binned value of 10^4^, 10^5^, 10^6^, or >10^7^ genomic copies/mL (35). Results were considered negative if the target quantity was less than 10^3–5^ copies/mL. For atypical bacterial or viral targets, results are reported dichotomously as either detected or not detected. Additionally, FilmArray-PN qualitatively identified genetic markers corresponding to bacterial targets, if they were present.

Targeted NGS was performed according to the RPIP protocol (Illumina, Inc., San Diego, CA, USA), including sample processing, library preparation, sequencing, bioinformatics analysis, interpretation, and comparison (36). Briefly, ribonucleic acids were extracted using the ALLPrep DNA/RNA Mini Kit (Qiagen, Hilden, Germany). A Qubit RNA HS Assay Kit (Thermo Fisher Scientific, Waltham, MA, USA) and a Nanodrop 2000 were used to evaluate the purity and concentration of the samples. Subsequently, the RNA was reverse transcribed to complementary deoxyribonucleic acid, which was later subjected to double-strand synthesis and labeled using the Illumina RNA Prep with Enrichment (L) Tagmentation Kit (Illumina). These fragments were then subjected to PCR for enrichment, and magnetic beads were used to screen for fragments of appropriate sizes. The length of the inserted fragments in the library was assessed using an Agilent 2100 Bioanalyzer, and their concentration was determined using a Qubit dsDNA HS Assay Kit (Thermo Fisher Scientific). RPIP (Illumina) with oligo probes was used to screen target fragments in the library, and the fragments of interest were amplified using PCR. A MiSeq Reagent Kit v3 (Illumina) was used for library purification and quantification. FastQC and MultiQC software were employed for quality control of the RPIP-generated readouts (37), and Trimmomatic was used to trim the adapter sequences. The RPIP reporting criteria for each species were designated based on a previous report.(12) Sequences with an alignment length of at least 75 nucleotides and 80% identity were selected for AMR analysis. The analysis includes 23 genotypes and 1,227 subtypes.

### Statistical analysis

Categorical variables were expressed as numbers with corresponding percentages, while continuous parameters were presented as mean ± standard deviation. Positive percent agreement (PPA) was defined as the proportion of positive results obtained by NGS/culture or FilmArray-PN/culture, while negative percent agreement (NPA) was defined as the proportion of negative outcomes rendered by NGS/culture or FilmArray-PN/culture. PPA and NPA are represented with 95% confidence intervals. Comparisons of positivity rates between NGS/culture and FilmArray-PN/culture were conducted using the Student’s *t*-test. Statistical significance was set at *P* ≤ 0.05.

## Data Availability

The data sets presented in this study will be available upon reasonable request to the corresponding author.

## SUPPLEMENTAL MATERIAL

Supplemental tables spectrum.02130-24-s0001.docx Tables S1 and S2.

ASM does not own the copyrights to Supplemental Material that may be linked to, or accessed through, an article. The authors have granted ASM a non-exclusive, world-wide license to publish the Supplemental Material files. Please contact the corresponding author directly for reuse.
