# Supplementary material for: Performance of a hybrid capture-based target enrichment next-generation sequencing for the identification of respiratory pathogens and resistance-associated genes in patients with severe pneumonia
Source: Microbiol Spectr. 2024 Nov 19;13(1):e02130-24. doi: 10.1128/spectrum.02130-24 (PMC11705961; doi:10.1128/spectrum.02130-24)
Supplement: Supplemental tables — Tables S1 and S2. [file spectrum.02130-24-s0001.docx]

**Performance of a Hybrid Capture-based Target Enrichment Next-generation Sequencing for the Identification of Respiratory Pathogens and Resistance-associated Genes in Patients with Severe Pneumonia**

Wei-Yu Hsu, Ting-Wei Kao, Hsin-Ching Cho, Sheng-Yuan Ruan, Tai-Fen Lee, Yu-Tsung Huang, Jung-Yien Chien, and TACTICS (TAiwan CollaboraTive Intensive Care Study) Group

**Supplementary table S1.** Modalities of specimen collection

| Modality | Number of patient |
| --- | --- |
| In-line suction | 60 (72.3%) |
| Bronchoalveolar lavage | 13 (15.7%) |
| Bronchial wash | 10 (12.0%) |

**Supplementary Table S2.** Detailed agreements of each pathogen between culture and RPIP / FilmArray-PN (by person, N=83)

|  | Culture vs. RPIP | | | | | | Culture vs. FilmArray-PN | | | | | |
| --- | --- | --- | --- | --- | --- | --- | --- | --- | --- | --- | --- | --- |
|  | Culture(+)  RPIP(+) | Culture(+)  RPIP(-) | Culture(-)  RPIP(+) | Culture(-)  RPIP(-) | PPA (%) | NPA (%) | Culture (+)  FilmArray (+) | Culture (+)  FilmArray (-) | Culture (-)  FilmArray (+) | Culture (-)  FilmArray (-) | PPA (%) | NPA (%) |
| *S. maltophilia* | 4 | 4 | 8 | 67 | 50.0 (15.7-84.3) | 84.3 (74.7-91.4) | 0 | 8 | 0 | 75 | 0.0 (0.0-36.9) | 100.0 (95.2-100.0) |
| *R. mucilaginosa* | 0 | 0 | 13 | 70 | - | 84.3 (74.7-91.4) | 0 | 0 | 0 | 83 | - | 100.0 (95.7-100.0) |
| *P. aeruginosa* | 7 | 1 | 4 | 71 | 87.5 (47.3-99.7) | 94.7 (86.9-98.5) | 8 | 0 | 5 | 70 | 100.0 (63.1-100.0) | 93.3 (85.1-97.8) |
| *B. cepacia complex* | 0 | 2 | 6 | 75 | 0.0 (0.0-84.2) | 92.6 (84.6-97.2) | 0 | 2 | 0 | 81 | 0.0 (0.0-84.2) | 100.0 (95.5-100.0) |
| *K. pneumoniae group*  *(K. variicola)* | 5 | 1 | 2 | 75 | 83.3 (35.9-99.6) | 98.7 (93.0-100.0) | 6 | 0 | 2 | 75 | 100.0 (54.1-100.0) | 97.4 (90.9-99.7) |
| *P. melaninogenica* | 0 | 0 | 7 | 76 | - | 91.6 (83.4-96.5) | 0 | 0 | 0 | 83 | - | 100.0 (95.7-100.0) |
| *S. aureus* | 4 | 1 | 2 | 76 | 80.0 (28.4-99.5) | 97.4 (91.0-99.7) | 5 | 1 | 4 | 73 | 83.3 (35.9-99.6) | 94.8 (87.2-98.6) |
| *V. parvula* | 0 | 0 | 7 | 76 | - | 94.0 (86.5-98.0) | 0 | 0 | 0 | 83 | - | 100.0 (95.7-100.0) |
| *E. faecium* | 0 | 0 | 5 | 78 | - | 94.0 (86.5-98.0) | 0 | 0 | 0 | 83 | - | 100.0 (95.7-100.0) |
| *P. fluorescens* | 0 | 0 | 5 | 78 | - | 95.2 (88.1-98.7) | 0 | 0 | 0 | 83 | - | 100.0 (95.7-100.0) |
| *C. striatum* | 0 | 0 | 4 | 79 | - | 96.4 (89.8-99.2) | 0 | 0 | 0 | 83 | - | 100.0 (95.7-100.0) |
| *A. odontolyticus* | 0 | 0 | 3 | 80 | - | 96.4 (89.8-99.2) | 0 | 0 | 0 | 83 | - | 100.0 (95.7-100.0) |
| *A. spp. (xylosoxidans)* | 2 | 0 | 1 | 80 | 100.0 (15.8-100.0) | 98.8 (93.3-100.0) | 0 | 2 | 0 | 81 | 0.0 (0.0-84.2) | 100.0 (95.5-100.0) |
| *G. haemolysans* | 0 | 0 | 3 | 80 | - | 96.4 (89.8-99.2) | 0 | 0 | 0 | 83 | - | 100.0 (95.7-100.0) |
| *H. influenzae* | 1 | 1 | 1 | 80 | 50.0 (1.3-98.7) | 100.0 (95.5-100.0) | 2 | 0 | 0 | 81 | 100.0 (15.8-100.0) | 100.0 (95.5-100.0) |
| *Ac-Ab complex* | 0 | 1 | 1 | 81 | 0.0 (0.0-97.5) | 98.8 (93.4-100.0) | 0 | 1 | 5 | 77 | - | 92.8 (84.9-97.3) |
| *A. graevenitzii* | 0 | 0 | 2 | 81 | - | 97.6 (91.6-99.7) | 0 | 0 | 0 | 83 | - | 100.0 (95.7-100.0) |
| *A. nosocomialis* | 2 | 1 | 1 | 79 | 66.7 (9.4-99.2) | 98.8 (93.2-100.0) | 0 | 1 | 0 | 82 | 0.0 (0.0-97.5) | 100.0 (95.6-100.0) |
| *E. meningoseptica* | 0 | 1 | 1 | 81 | 0.0 (0.0-97.5) | 98.8 (93.4-100.0) | 0 | 1 | 0 | 82 | 0.0 (0.0-97.5) | 100.0 (95.6-100.0) |
| *M. catarrhalis* | 0 | 1 | 1 | 81 | 0.0 (0.0-97.5) | 98.8 (93.4-100.0) | 1 | 0 | 0 | 82 | 100.0 (2.5-100.0) | 100.0 (95.6-100.0) |
| *M. osloensis* | 0 | 0 | 2 | 81 | - | 97.6 (91.6-99.7) | 0 | 0 | 0 | 83 | - | 100.0 (95.7-100.0) |
| *S. constellatus* | 0 | 0 | 2 | 81 | - | 97.6 (91.6-99.7) | 0 | 0 | 0 | 83 | - | 100.0 (95.7-100.0) |
| *A. pittii* | 0 | 0 | 1 | 82 | - | 98.8 (93.5-100.0) | 0 | 0 | 0 | 83 | - | 100.0 (95.7-100.0) |
| *C. gingivalis* | 0 | 0 | 1 | 82 | - | 98.8 (93.5-100.0) | 0 | 0 | 0 | 83 | - | 100.0 (95.7-100.0) |
| *D. acidovorans* | 1 | 0 | 0 | 82 | 100.0 (2.5-100.0) | 100.0 (95.6-100.0) | 0 | 1 | 0 | 82 | 0.0 (0.0-97.5) | 100.0 (95.6-100.0) |
| *D. pneumosintes* | 0 | 0 | 1 | 82 | - | 98.8 (93.5-100.0) | 0 | 0 | 0 | 83 | - | 100.0 (95.7-100.0) |
| *E. cloacae complex* | 0 | 0 | 1 | 82 | - | 98.8 (93.5-100.0) | 0 | 0 | 2 | 81 | - | 97.6 (91.6-99.7) |
| *E. coli* | 1 | 0 | 0 | 82 | 100.0 (2.5-100.0) | 100.0 (95.6-100.0) | 1 | 0 | 0 | 82 | 100.0 (2.5-100.0) | 100.0 (95.6-100.0) |
| *E. faecalis* | 0 | 0 | 1 | 82 | - | 98.8 (93.5-100.0) | 0 | 0 | 0 | 83 | - | 100.0 (95.7-100.0) |
| *F. nucleatum* | 0 | 0 | 1 | 82 | - | 98.8 (93.5-100.0) | 0 | 0 | 0 | 83 | - | 100.0 (95.7-100.0) |
| *H. haemolyticus* | 0 | 0 | 1 | 82 | - | 98.8 (93.5-100.0) | 0 | 0 | 0 | 83 | - | 100.0 (95.7-100.0) |
| *H. parainfluenzae* | 0 | 0 | 1 | 82 | - | 98.8 (93.5-100.0) | 0 | 0 | 0 | 83 | - | 100.0 (95.7-100.0) |
| *K. aerogenes* | 0 | 1 | 0 | 82 | 0.0 (0.0-97.5) | 100.0 (95.6-100.0) | 0 | 1 | 0 | 82 | 0.0 (0.0-97.5) | 100.0 (95.6-100.0) |
| *N. flavescens* | 0 | 0 | 1 | 82 | - | 98.8 (93.5-100.0) | 0 | 0 | 0 | 83 | - | 100.0 (95.7-100.0) |
| *P. intermedia* | 0 | 0 | 1 | 82 | - | 98.8 (93.5-100.0) | 0 | 0 | 0 | 83 | - | 100.0 (95.7-100.0) |
| *Proteus spp.* | 1 | 0 | 0 | 82 | 100.0 (2.5-100.0) | 100.0 (95.6-100.0) | 1 | 0 | 1 | 81 | 100.0 (2.5-100.0) | 97.6 (91.6-99.7) |
| *S. anginosus* | 0 | 0 | 1 | 82 | - | 98.8 (93.5-100.0) | 0 | 0 | 0 | 83 | - | 100.0 (95.7-100.0) |
| *S. parapaucimobilis* | 0 | 1 | 0 | 82 | 0.0 (0.0-97.5) | 100.0 (95.6-100.0) | 0 | 1 | 0 | 82 | 0.0 (0.0-97.5) | 100.0 (95.6-100.0) |
| *S. marcescens* | 0 | 0 | 1 | 82 | - | 98.8 (93.5-100.0) | 0 | 0 | 2 | 81 | - | 97.6 (91.6-99.7) |
| *S. mitis* | 0 | 0 | 1 | 82 | - | 98.8 (93.5-100.0) | 0 | 0 | 0 | 83 | - | 100.0 (95.7-100.0) |
| *S. paucimobilis* | 0 | 0 | 1 | 82 | - | 98.8 (93.5-100.0) | 0 | 0 | 0 | 83 | - | 100.0 (95.7-100.0) |
| *T. denticola* | 0 | 0 | 1 | 82 | - | 98.8 (93.5-100.0) | 0 | 0 | 0 | 83 | - | 100.0 (95.7-100.0) |
| *S. agalactiae* | 0 | 0 | 0 | 83 | - | 100.0 (95.7-100.0) | 0 | 0 | 2 | 81 | - | 97.6 (91.6-99.7) |
| *C. pneumoniae* | 0 | 0 | 0 | 83 | - | 100.0 (95.7-100.0) | 0 | 0 | 0 | 83 | - | 100.0 (95.7-100.0) |
| *L. pnemophlia* | 0 | 0 | 0 | 83 | - | 100.0 (95.7-100.0) | 0 | 0 | 1 | 82 | - | 98.8 (93.5-100.0) |
| *M. pneumoniae* | 0 | 0 | 0 | 83 | - | 100.0 (95.7-100.0) | 0 | 0 | 0 | 83 | - | 100.0 (95.7-100.0) |
| *HSV1* | 0 | 0 | 16 | 67 | - | 80.7 (70.6-88.6) | 0 | 0 | 0 | 83 | - | 100.0 (95.7-100.0) |
| *CMV* | 0 | 0 | 15 | 68 | - | 81.9 (72.0-89.5) | 0 | 0 | 0 | 83 | - | 100.0 (95.7-100.0) |
| *EBV* | 0 | 0 | 14 | 69 | - | 83.1 (73.3-90.5) | 0 | 0 | 0 | 83 | - | 100.0 (95.7-100.0) |
| *CoV* | 0 | 0 | 12 | 71 | - | 85.5 (76.1-92.3) | 0 | 0 | 0 | 83 | - | 100.0 (95.7-100.0) |
| *HHV6* | 0 | 0 | 4 | 79 | - | 95.2 (88.1-98.7) | 0 | 0 | 0 | 83 | - | 100.0 (95.7-100.0) |
| *Human Rhinovirus/Enterovirus* | 0 | 0 | 2 | 81 | - | 97.6 (91.6-99.7) | 0 | 0 | 4 | 79 | - | 95.2 (88.1-98.7) |
| *Adenovirus* | 0 | 0 | 1 | 82 | - | 98.8 (93.5-100.0) | 0 | 0 | 3 | 80 | - | 96.4 (89.8-99.2) |
| *Parainfluenzae Virus* | 0 | 0 | 1 | 82 | - | 98.8 (93.5-100.0) | 0 | 0 | 2 | 81 | - | 97.6 (91.6-99.7) |
| *Human Metapneumovirus* | 0 | 0 | 0 | 83 | - | 100.0 (95.7-100.0) | 0 | 0 | 3 | 80 | - | 96.4 (89.8-99.2) |
| *Influenzae A* | 0 | 0 | 0 | 83 | - | 100.0 (95.7-100.0) | 0 | 0 | 2 | 81 | - | 97.6 (91.6-99.7) |
| *Pneumocystis jirovecii* | 0 | 0 | 7 | 76 | - | 91.6 (83.4-96.5) | 0 | 0 | 0 | 83 | - | 100.0 (95.7-100.0) |
| *Fusarium solani* | 0 | 0 | 2 | 81 | - | 97.6 (91.6-99.7) | 0 | 0 | 0 | 83 | - | 100.0 (95.7-100.0) |
| *Aspergillus flavus* | 0 | 0 | 1 | 82 | - | 98.8 (93.5-100.0) | 0 | 0 | 0 | 83 | - | 100.0 (95.7-100.0) |
| *Aspergillus versicolor* | 0 | 0 | 1 | 82 | - | 98.8 (93.5-100.0) | 0 | 0 | 0 | 83 | - | 100.0 (95.7-100.0) |

RPIP: Respiratory Pathogen ID/AMR Enrichment kit; FilmArray-PN: Biofire FilmArray Pneumonia Panel
